# Supplementary material for: Extended coverage of human serum glycosphingolipidome by 4D-RP-LC TIMS-PASEF unravels association with Parkinson’s disease
Source: Nat Commun. 2025 May 16;16:4567. doi: 10.1038/s41467-025-59755-6 (PMC12084332; doi:10.1038/s41467-025-59755-6)
Supplement: Supplementary file 1 — Supplementary Information [file 41467_2025_59755_MOESM1_ESM.pdf]

Supplementary information

Extended coverage of human serum glycosphingolipidome by 4D-RP-LC TIMS-PASEF unravels association with Parkinson's disease

Huong Giang Vo[1], Gabriel Gonzalez-Escamilla[2,3], Daniela Mirzac[2], Lilia Rotaru[4], Damian Herz[2], Sergiu Groppa[2,3] and Laura Bindila[1]

1. Clinical Lipidomics Unit, Institute of Physiological Chemistry, University Medical Center, Duesbergweg 6, 55128 Mainz, Germany
2. Movement Disorders, Imaging and Neurostimulation, Department of Neurology, University Medical Center, Langenbeckstr. 1, 55131 Mainz, Germany
3. Department of Neurology, Saarland University, Saarland University Hospital, Kirrberger Str 100, 66421 Homburg, Germany
4. Laboratory of Functional Neurology, Diomid Gherman Institute of Neurology and Neurosurgery, VI. Korolenko St 2, Chisinau, Republic of Moldova

* Corresponding author: Laura Bindila, PhD, Email: bindila@uni-mainz.de

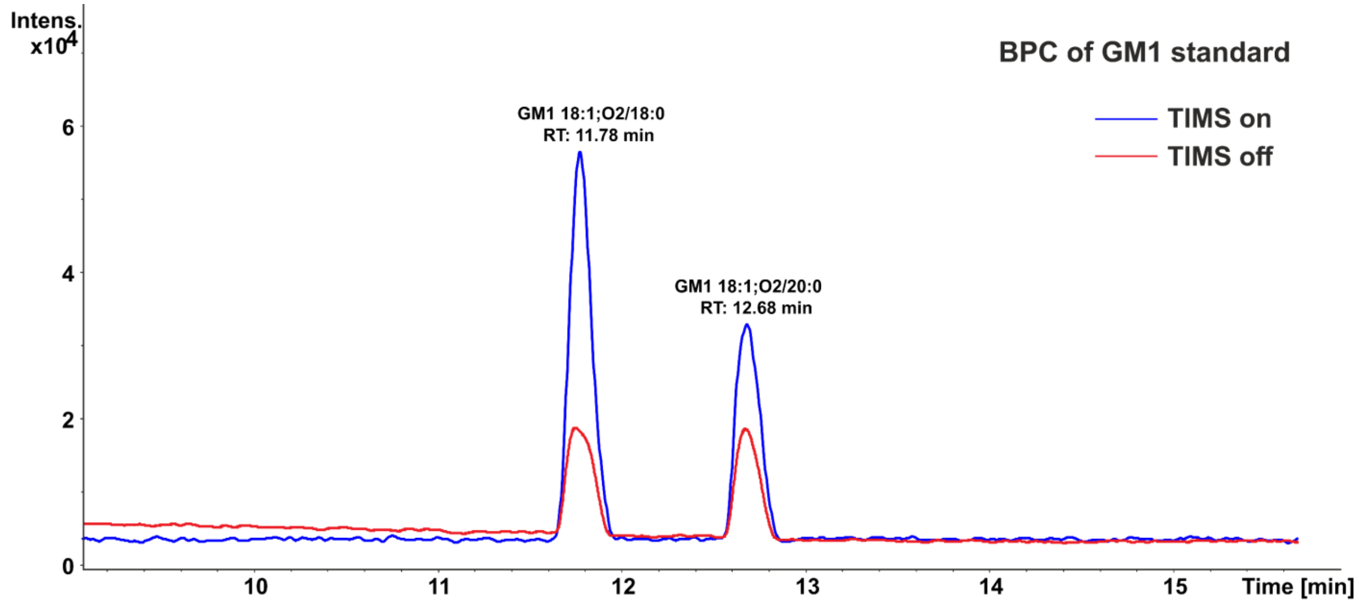

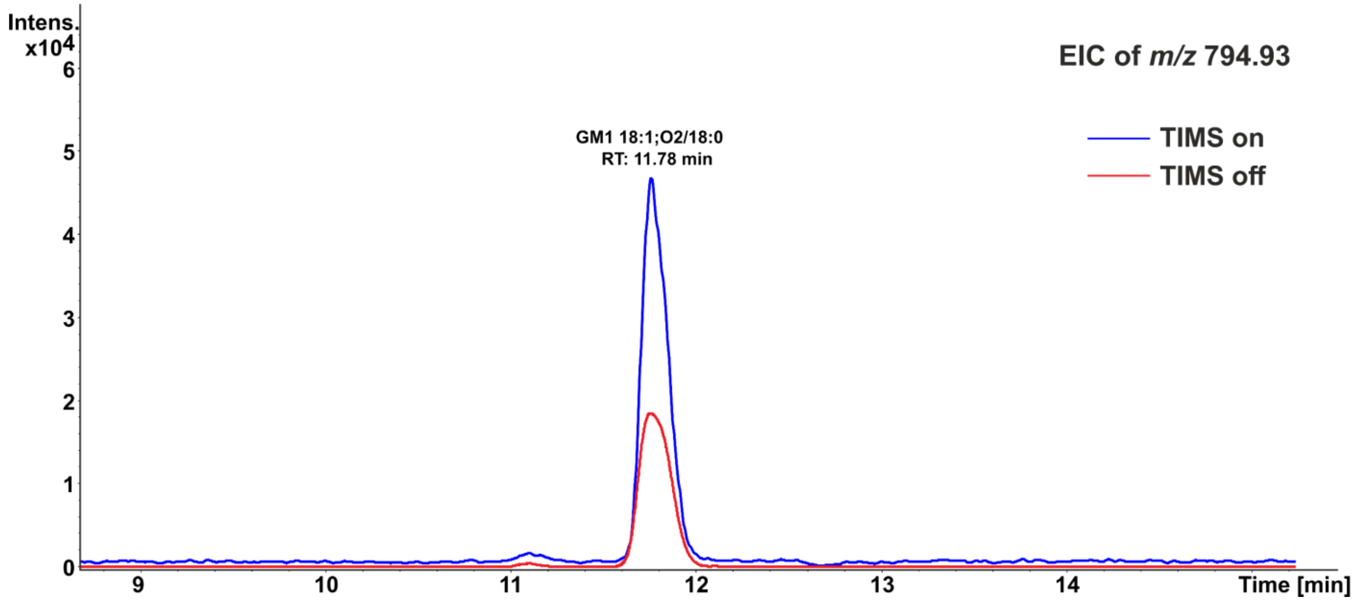

Supplementary Figure 1. Base peak chromatogram (BPC) of GM1 standard and extracted-ion chromatogram (EIC) of GM1 18:1;O2/18:0 with TIMS on/off. GM1 standard was prepared at 2pmol/µL and 20 µL of the working aliquot was injected for each measurement. The measurements were performed with TIMS in PASEF mode (TIMS on) and MS-only mode (TIMS off). Figure demonstrates that when TIMS is on, both the BPC and EIC signals for the GM1 standard are notably higher than those observed in TIMS off, indicating improved signal intensity and sensitivity with TIMS in PASEF mode.

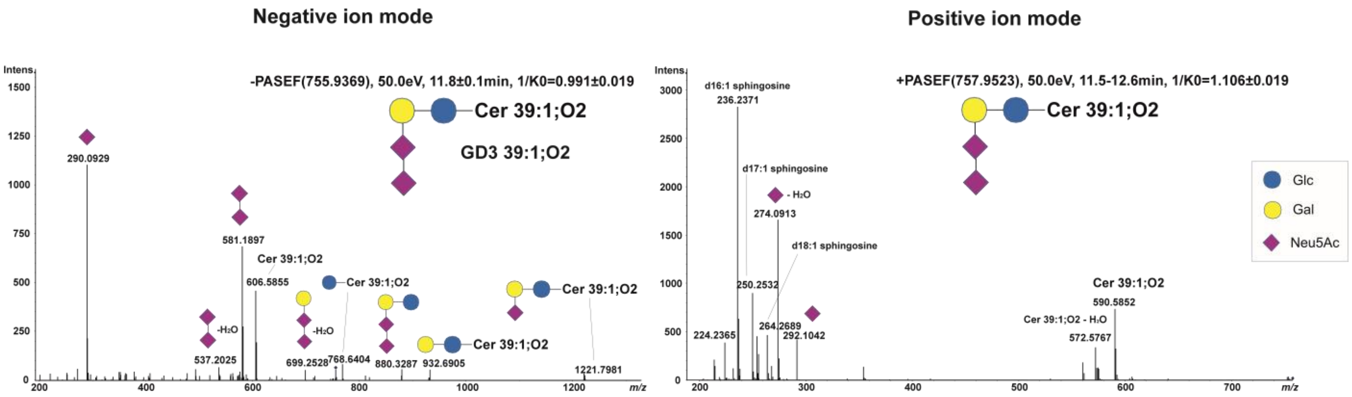

Supplementary Figure 2. PASEF spectra of GD3 39:1;O2 in negative ion mode at CE 50 eV and positive ion mode at CE 50eV. GD3 39:1;O2 was identified in customized standard mixture, consisting of GM4, GM3, GM2, GM1, GD3, GD2, GD1a, GD1b, GT1b, and GQ1b at 5 pmol/µL. In negative ion mode, precursor ions with fragment at nominal *m/z* 290 in their MS/MS spectra were filtered manually structurally annotated, and elucidated based on their MS/MS spectra. Similarly, in positive ion mode, precursor ions with fragment at *m/z* 274 were analysed, enabling sphingoid base identification, including fragments *m/z* 236 for 16:1;O2, *m/z* 250 for 17:1;O2, and *m/z* 264 for 18:1;O2. Blue circle represents Glucose, yellow circle represents Galactose, and purple diamond represents *N*-acetylneuraminic acid.

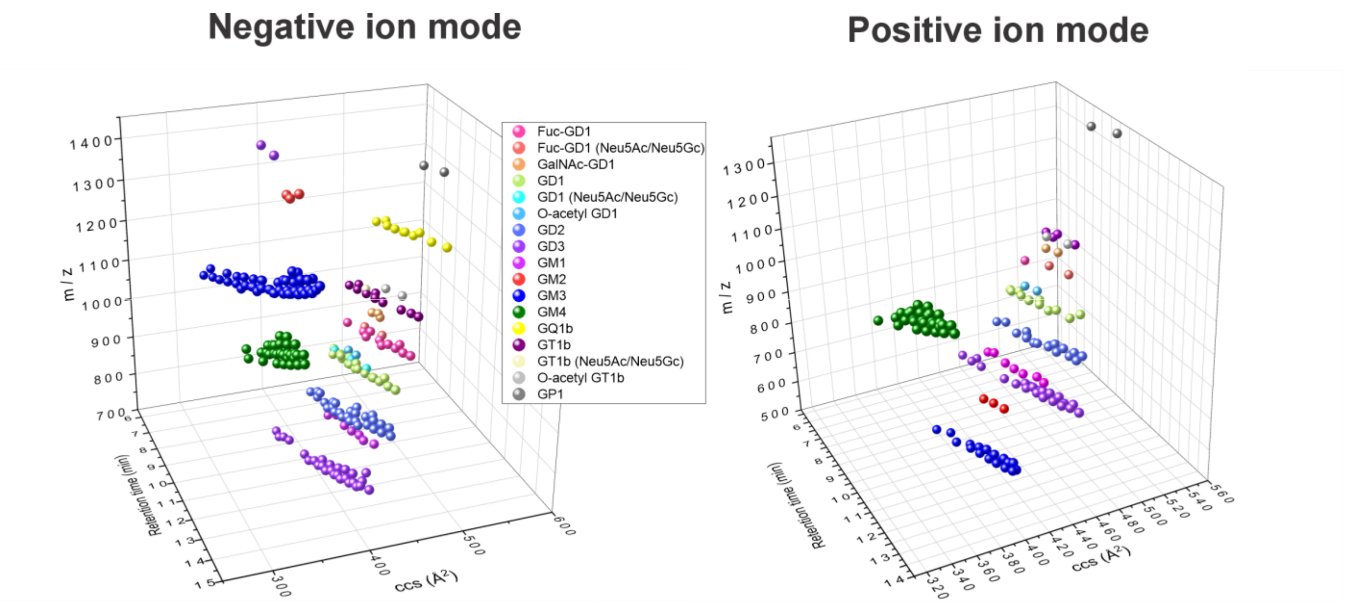

Supplementary Figure 3. Three-dimension (*m/z*, CCS, retention time (RT)) distribution of GSLs in customized standard mixture (average value of each descriptor from n = 3 measurements). Customized standard mixture, containing GM4, GM3, GM2, GM1, GD3, GD2, GD1a, GD1b, GT1b, and GQ1b, was prepared at 5 pmol/µL. For analysis, 100 pmol of each standard was injected on the column of the RP-UHPLC TIMS-MS system. The figure illustrates the 3D distribution of gangliosides analyzed in both negative and positive ion modes, with each subclass occupying distinct regions within the 3D space. Detailed descriptors are provided in Supplementary Data 1.

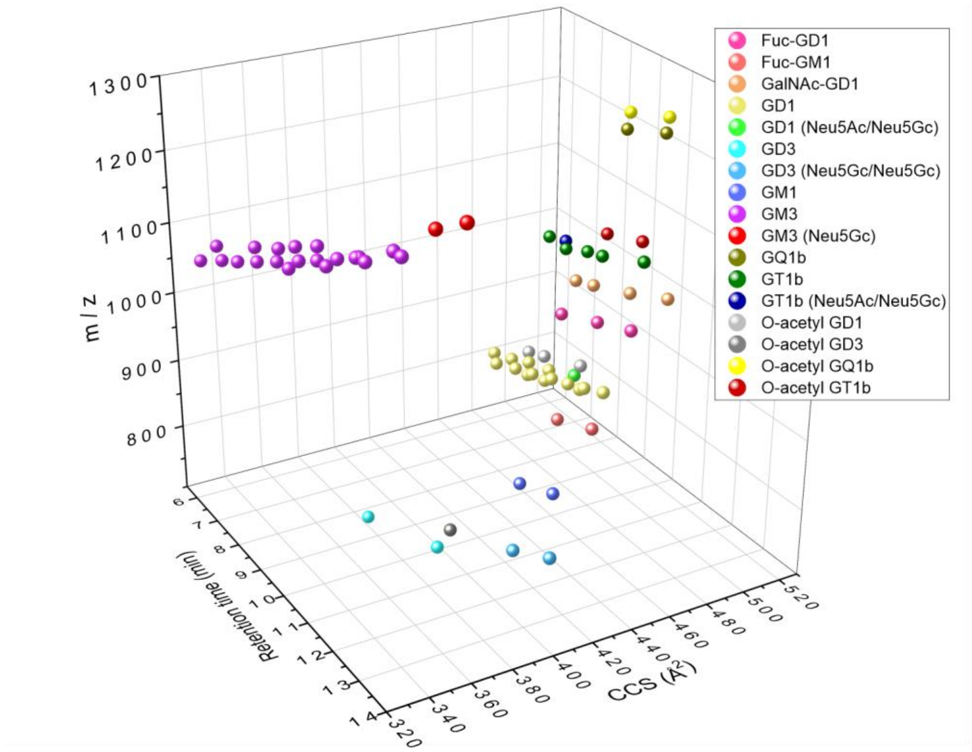

Supplementary Figure 4. Three-dimension (*m/z*, CCS, RT) distribution of GSLs in porcine brain extraction (average value of each descriptor from n = 3 measurements). Porcine brain extract (Product No. 860053) was obtained from Avanti Polar Lipids. The extract was dissolved in MeOH/water (8:2, v/v) to achieve a final concentration of 5 pmol/µL, with 20µL injected onto the column of the RP-UHPLC TIMS-MS system for the measurement. The measurement was conducted in negative ion mode, with the collision energy for PASEF set to 50 eV. The figure illustrates the 3D (*m/z*, CCS, and RT) distribution of gangliosides analyzed in negative ion mode, with each subclass occupying distinct regions within the 3D space. Detailed descriptors are provided in Supplementary Table 3.

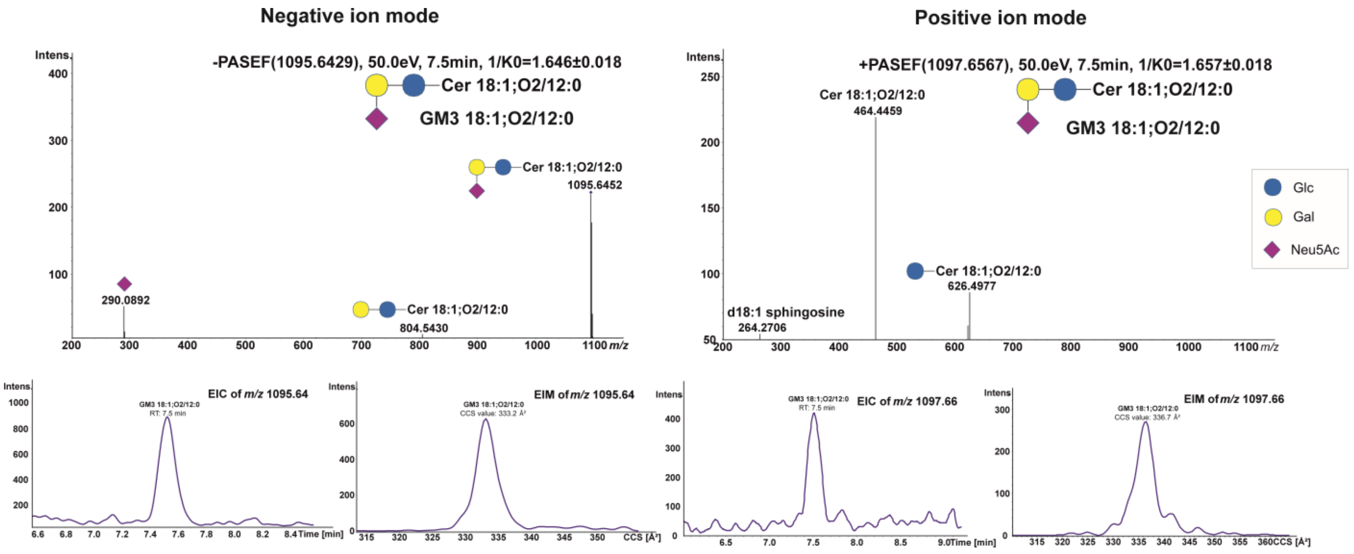

Supplementary Figure 5. GM3 18:1;O2/12:0 in human serum sample. For the analysis, 20 µL of fraction 1, corresponding to 120 µL serum was inject on the column of the RP-UHPLC TIMS-MS system. Measurements were performed both negative and positive ion modes with CE at 50 eV. Structural characterization of GM3 18:1;O2/12:0 was performed by manually curating the PASEF spectra and evaluating the EIC and EIM in both ionization modes. Blue circle represents Glucose, yellow circle represents Galactose, and purple diamond represents *N*-acetylneuraminic acid.

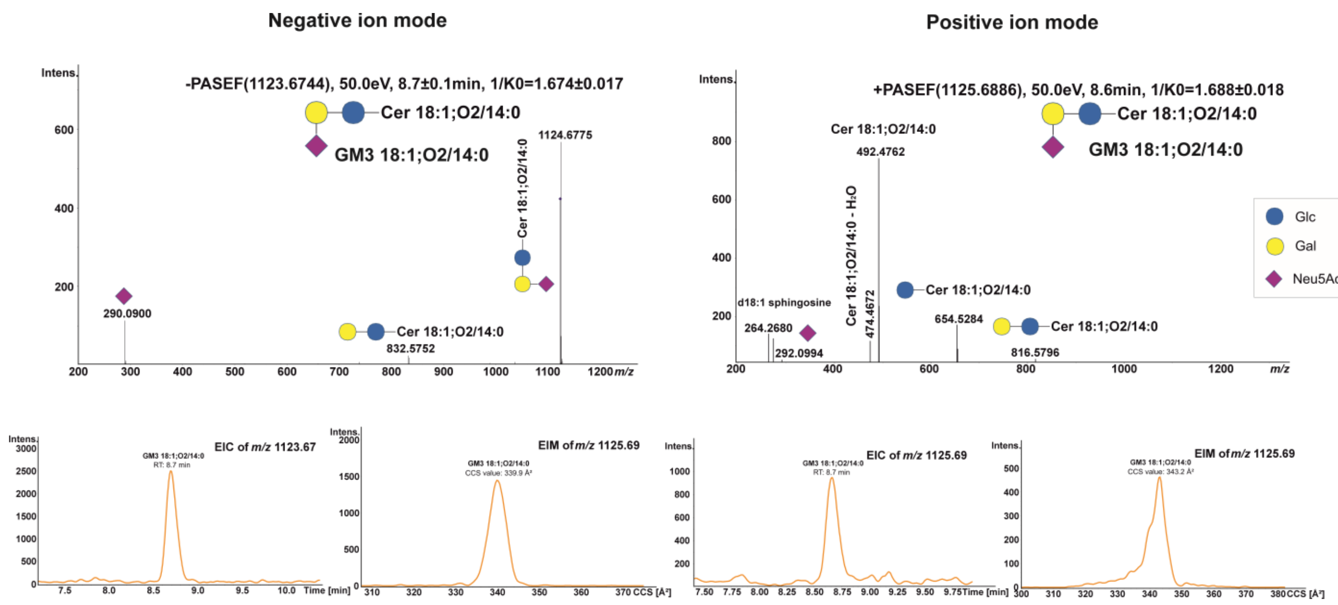

Supplementary Figure 6. GM3 18:1;O2/14:0 in human serum sample. For the analysis, 20 µL of fraction 1, corresponding to 120 µL serum was inject on the column of the RP-UHPLC TIMS-MS system. Measurements were performed both negative and positive ion modes with CE at 50 eV. Structural characterization of GM3 18:1;O2/14:0 was performed by manually curating the PASEF spectra and evaluating the EIC and EIM in both ionization modes. Blue circle represents Glucose, yellow circle represents Galactose, and purple diamond represents *N*-acetylneuraminic acid.

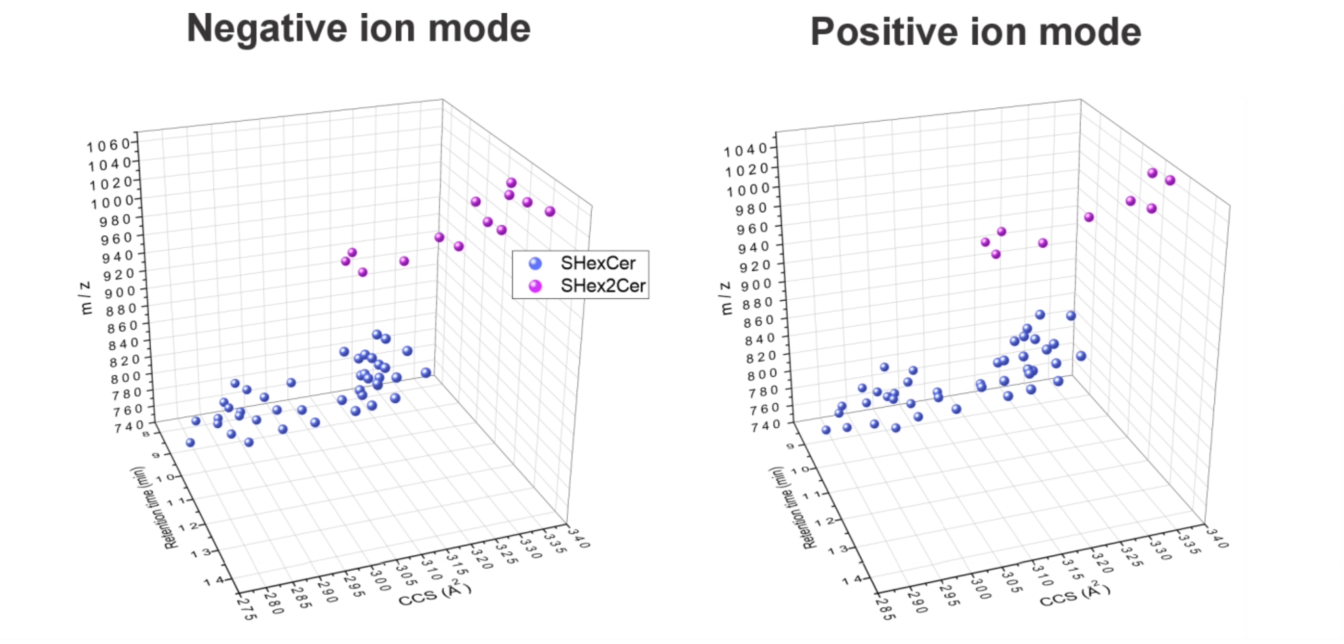

Supplementary Figure 7. Three-dimension (*m/z*, CCS, RT) distribution of sulfatides in human serum in fraction 1 (average value of each descriptor from n = 3 measurements). For the analysis, 20 µL of fraction 1, corresponding to 120 µL serum was inject on the column of the RP-UHPLC TIMS-MS system. Measurements were performed both negative and positive ion modes with CE at 50 eV. Sulfatides showed better ionization in negative ion mode, though fragmentation at CE 50eV was poor. PASEF spectra at positive ion mode was used to confirm the sulfatide structures. Figure illustrates highlights the distinct 3D (m/z, CCS, RT) space of sulfatide subclasses in negative and positive ion modes. Detailed descriptors are provided in Supplementary Data 6.

Supplementary Table 1. Ganglioside standards in customized standard mixture. Customized standard mixture was prepare at the concentration of 5 pmol/µL.

No	Name	Source	Manufacturer
**1**	Ganglioside GM4	Human brain	Calbiochem
**2**	Ganglioside GM3	Bovine milk	Avanti Polar Lipids
**3**	Ganglioside GM2	Bovine brain	Avanti Polar Lipids
**4**	Ganglioside GM1	Ovine brain	Avanti Polar Lipids
**5**	Ganglioside GD3	Bovine milk	Avanti Polar Lipids
**6**	Ganglioside GD2	Human brain	Calbiochem
**7**	Ganglioside GD1a	Porcine brain	Avanti Polar Lipids
**8**	Ganglioside GD1b	Porcine brain	Avanti Polar Lipids
**9**	Ganglioside GT1b	Porcine brain	Avanti Polar Lipids
**10**	Ganglioside GQ1b	Porcine brain	Avanti Polar Lipids

Supplementary Table 2. Identified gangliosides in total ganglioside in porcine brain extract. Porcine brain extract (Product No. 860053) was obtained from Avanti Polar Lipids. The extract was dissolved in MeOH/water (8:2, v/v) to achieve a final concentration of 5 pmol/µL. 100 pmol porcine brain extract was injected on the column of the RP-UHPLC TIMS-MS system for the measurement. The measurement was conducted in negative ion mode, with the collision energy for PASEF set to 50 eV. The table shows the 3D parameters (*m/z*, retention time, and CCS value) of each gangliosides identified in porcine brain extract.

RT [min]	CCS (Å²)	*m/z* theor.	*m/z* meas.	ppm	Ion type	Name	Molecular Formula
11	470.68	1005.5312	1005.5274	3.77	[M-2H]2-	Fuc-GD1 18:0;O2/20:0	C92H164N4O43
9.66	468.7	990.5077	990.5069	0.83	[M-2H]2-	Fuc-GD1 18:1;O2/18:0	C90H158N4O43
10.64	472.5	1004.5234	1004.5215	1.86	[M-2H]2-	Fuc-GD1 18:1;O2/20:0	C92H162N4O43
11.58	475	1018.5390	1018.5383	0.71	[M-2H]2-	Fuc-GD1 18:1;O2/22:0	C94H166N4O43
10.39	456.2	867.9627	867.9628	-0.06	[M+HCOOH-2H]2-	Fuc-GM1 18:1;O2/18:0	C79H141N3O35
11.33	460	881.9784	881.978	0.45	[M+HCOOH-2H]2-	Fuc-GM1 18:1;O2/20:0	C81H145N3O35
9.62	486.5	1019.0185	1019.0087	9.57	[M-2H]2-	GalNAc-GD1 18:1;O2/18:0	C92H161N5O44
10.61	490.2	1033.0341	1033.0324	1.65	[M-2H]2-	GalNAc-GD1 18:1;O2/20:0	C94H165N5O44
11.51	495.3	1047.0498	1047.0466	3.01	[M-2H]2-	GalNAc-GD1 18:1;O2/22:0	C96H169N5O44
10.11	453.3	918.4866	918.4845	2.27	[M-2H]2-	GD1 18:0;O2/18:0	C84H150N4O39
11.06	457.2	932.5022	932.4998	2.62	[M-2H]2-	GD1 18:0;O2/20:0	C86H154N4O39
8.7	448.1	903.4631	903.462	1.23	[M-2H]2-	GD1 18:1;O2/16:0	C82H144N4O39
9.75	452	917.4788	917.4781	0.72	[M-2H]2-	GD1 18:1;O2/18:0	C84H148N4O39
9	452	916.4709	916.4698	1.24	[M-2H]2-	GD1 18:1;O2/18:1	C84H146N4O39
10.26	454.6	924.4866	924.486	0.64	[M-2H]2-	GD1 18:1;O2/19:0	C85H150N4O39
9.45	454.6	923.4788	923.4776	1.26	[M-2H]2-	GD1 18:1;O2/19:1	C85H148N4O39
10.74	455.9	931.4944	931.4941	0.34	[M-2H]2-	GD1 18:1;O2/20:0	C86H152N4O39
10.08	455.9	930.4866	930.4879	-1.41	[M-2H]2-	GD1 18:1;O2/20:1	C86H150N4O39
11.2	457.2	938.5022	938.5014	0.89	[M-2H]2-	GD1 18:1;O2/21:0	C87H154N4O39
11.67	459.8	945.5101	945.5098	0.28	[M-2H]2-	GD1 18:1;O2/22:0	C88H156N4O39
9.7	456.87	925.4762	925.4755	0.78	[M-2H]2-	GD1 (Neu5Ac/Neu5Gc) 18:1;O2/18:0	C84H148N4O40
10.7	459.8	939.4919	939.4905	1.46	[M-2H]2-	GD1 (Neu5Ac/Neu5Gc) 18:1;O2/20:0	C86H152N4O40
10.52	391.1	734.9127	734.9138	-1.55	[M-2H]2-	GD3 18:1;O2/18:0	C70H125N3O29
12.17	406.6	778.9389	778.9383	0.74	[M-2H]2-	GD3 (Neu5Gc/Neu5Gc) 18:1;O2/22:0	C74H133N3O31
12.98	413.1	792.9545	792.9511	4.33	[M-2H]2-	GD3 (Neu5Gc/Neu5Gc) 18:1;O2/24:0	C76H137N3O31
10.54	434.2	794.9338	794.9329	1.13	[M+HCOOH-2H]2-	GM1 18:1;O2/18:0	C73H131N3O31
11.45	438	808.9494	808.9491	0.43	[M+HCOOH-2H]2-	GM1 18:1;O2/20:0	C75H135N3O31
10.97	349.6	1179.7372	1179.7357	1.26	[M-H]-	GM3 18:1;O2/18:0	C59H108N2O21
11.83	354.5	1207.7685	1207.7666	1.56	[M-H]-	GM3 18:1;O2/20:0	C61H112N2O21
12.67	362.4	1251.7947	1251.7903	3.52	[M-H]-	GM3 (Neu5Gc) 18:1;O2/22:0	C63H116N2O22
13.51	364.7	1279.8260	1279.8257	0.24	[M-H]-	GM3 (Neu5Gc) 18:1;O2/24:0	C65H120N2O22
9	512.7	1208.5742	1208.573	0.98	[M-2H]2-	GQ1b 18:1;O2/18:0	C106H182N6O55
9.97	517.7	1222.5898	1222.5882	1.34	[M-2H]2-	GQ1b 18:1;O2/20:0	C108H186N6O55
9.13	478.7	1063.0265	1063.026	0.45	[M-2H]2-	GT1b 18:1;O2/18:0	C95H165N5O47
8.46	480	1062.0186	1062.0172	1.36	[M-2H]2-	GT1b 18:1;O2/18:1	C95H163N5O47
9.63	482.5	1070.0343	1070.0331	1.12	[M-2H]2-	GT1b 18:1;O2/19:0	C96H167N5O47
10.13	482.5	1077.0421	1077.0409	1.14	[M-2H]2-	GT1b 18:1;O2/20:0	C97H169N5O47
11.1	488.8	1091.0578	1091.0536	3.83	[M-2H]2-	GT1b 18:1;O2/22:0	C99H173N5O47
9.04	479.9	1071.0239	1071.0242	-0.25	[M-2H]2-	GT1b (Neu5Ac/Neu5Gc) 18:1;O2/18:0	C95H165N5O48
10.1	484.54	1085.0396	1085.0372	2.19	[M-2H]2-	GT1b (Neu5Ac/Neu5Gc) 18:1;O2/20:0	C97H169N5O48
9.74	458.5	938.4840	938.4831	1.01	[M-2H]2-	O-acetyl GD1 18:1;O2/18:0	C86H150N4O40
10.74	462.4	952.4997	952.5007	-1.05	[M-2H]2-	O-acetyl GD1 18:1;O2/20:0	C88H154N4O40
10.54	397.6	755.9179	755.9188	-1.13	[M-2H]2-	O-acetyl GD3 18:1;O2/18:0	C72H127N3O30
8.95	515.1	1229.5795	1229.5771	1.92	[M-2H]2-	O-acetyl GQ1b 18:1;O2/18:0	C108H184N6O56
9.98	518.9	1243.5951	1243.5922	2.34	[M-2H]2-	O-acetyl GQ1b 18:1;O2/20:0	C110H188N6O56
9.5	495.2	1084.0318	1084.0316	0.14	[M-2H]2-	O-acetyl GT1b 18:1;O2/18:0	C97H167N5O48
10.51	497.7	1098.0474	1098.0389	7.75	[M-2H]2-	O-acetyl GT1b 18:1;O2/20:0	C99H171N5O48

Supplementary Table 3. Evaluation of the carry-over effect of 5 pmol/µL customized standard mixture. Customized standard mixture, containing GM4, GM3, GM2, GM1, GD3, GD2, GD1a, GD1b, GT1b, and GQ1b, was prepared at a concentration of 5 pmol/µL. For analysis, 100 pmol of each standard and 50 ng of each internal standard were injected on the column of the RP-UHPLC TIMS-MS system for the measurement. The carry-over effect of the most abundant analyte in each standard was assessed by analyzing a blank sample following the injection of the highest concentration of the customized standard mixture. The peak areas of analytes in the standard mixture and blank sample were compared to determine the carry-over. The average carry-over effect across all analytes and internal standards was determined to be 1.2% with the highest carry-over observed in GD3 18:1;O2/18:0-d3 at 5.79%.

Name	Peak area in Std mix	Peak area in Blank	Carry-over effect (%)
**GD1 18:1;O2/18:0**	6233911	138379	2.22
**GD2 18:1;O2/20:0**	1959542	60005	3.06
**GD3 16:1;O2/23:0**	191862	5943	3.1
**GD3 18:1;O2/18:0-d3**	199686	11571	5.79
**GM1 18:1;O2/18:0**	547683	5974	1.09
**GM1 18:1;O2/18:0-d5**	108552	0	0
**GM2 18:1;O2/16:0-d9**	193125	0	0
**GM2 18:1;O2/18:0**	479591	0	0
**GM3 18:1;O2/18:0-d5**	1129134	8890	0.79
**GM3 18:1;O2/22:0**	630450	0	0
**GM4 18:1;O2/24:0;O**	1218053	5886	0.48
**GQ1b 18:1;O2/18:0**	477557	0	0
**GT1b 18:1;O2/18:0**	2417734	0	0

Supplementary Table 4. Evaluation of the carry-over effect of sialylated glycosphingolipid in human serum extract. For the analysis, 20 µL of fraction 2, corresponding to 120 µL serum was inject on the column of the RP-UHPLC TIMS-MS system. The carry-over effect of the most abundant sialylated GSL in each subclass was assessed by analyzing a blank sample injected after three extract replicates of serum extraction. The average peak areas of analyte in three replicate extracts of fraction 2 and blank sample were compared to determine the carry-over, revealing no carry-over effect for sialylated GSLs in serum during the measurement.

Name	Average peak area in 3 replicates in Fraction 2	Peak area in Blank	Carry-over effect (%)
**GD1 18:1;O2/18:0**	37749	0	0
**GD2 18:1;O2/16:0**	5104	0	0
**GD3 18:1;O2/18:0-d3**	11462	0	0
**GD3 18:1;O2/20:0**	27443	0	0
**GM1 18:1;O2/18:0-d5**	9507	0	0
**GM2 18:1;O2/16:0-d9**	41668	0	0
**GM2 18:1;O2/18:0**	3668	0	0
**GM3 18:1;O2/16:0**	533686	0	0
**GM3 18:1;O2/18:0-d5**	186999	0	0
**GT1b 18:1;O2/18:0**	15436	0	0
**Neu5Ac-Fuc-nLc10Cer 18:1;O2/16:0**	2629	0	0
**Neu5Ac-Fuc-nLc6Cer 18:1;O2/16:0**	1684	0	0
**Neu5Ac-Fuc-nLc8Cer 18:1;O2/16:0**	3745	0	0
**Neu5Ac-nLc4Cer 18:1;O2/16:0**	8278	0	0
**Neu5Ac-nLc6Cer 18:1;O2/16:0**	16560	0	0
**Neu5Ac-nLc8Cer 18:1;O2/16:0**	6432	0	0
**O-acetyl GD1 18:1;O2/18:0**	3635	0	0
