# Supplementary material for: Extended coverage of human serum glycosphingolipidome by 4D-RP-LC TIMS-PASEF unravels association with Parkinson’s disease
Source: Nat Commun. 2025 May 16;16:4567. doi: 10.1038/s41467-025-59755-6 (PMC12084332; doi:10.1038/s41467-025-59755-6)
Supplement: Supplementary file 2 — Description of Additional Supplementary Files [file 41467_2025_59755_MOESM2_ESM.pdf]

Description of Additional Supplementary Files

File Name: Supplementary Data 1

Description: List of gangliosides and their 3D-descriptors (*m/z*, retention time (RT), collisional cross section (CCS)) from customized standard mixtures. Customized standard mixture, containing GM4, GM3, GM2, GM1, GD3, GD2, GD1a, GD1b, GT1b, and GQ1b, was prepared at a concentration of 5 pmol/µL. For analysis, 100 pmol of each standard and 50 ng of each internal standard were injected on the column of the RP-LC-TIMS-MS system for the measurement. Standard mixtures were measured in both negative ion mode and positive ion mode at collision energy 50 eV. Positive mode was used for the identification of sphingoid base and *N*-linked fatty acyl chain of gangliosides. Spectral library is deposited in the MASS Spectrometry Interactive Virtual Environment (MassIVE) under the identifier MSV000097015 [https://dx.doi.org/10.25345/C53J39C93]. The spectral library generated and used in this study is additionally available under the following link: Research Tools [https://www.unimedizin-mainz.de/lipidomics-unit/lipid-research/research-tools.html].

File Name: Supplementary Data 2

Description: A comprehensive list of 147 sialylated glycosphingolipids (GSLs) along with their 3D-descriptors (*m/z*, CCS, RT), identified from a volunteer's serum in a two-fraction extract. This dataset was used to evaluate the coverage of sialylated GSLs in 300 µL human serum, employing a silica-fractionation method that separate the extract into two or four fractions for analysis.

File Name: Supplementary Data 3

Description: A comprehensive list of 135 sialylated glycosphingolipids (GSLs) along with their 3D-descriptors (*m/z*, RT, CCS), identified from a volunteer's serum in a four-fraction extract. This dataset was used to evaluate the coverage of sialylated GSLs in 300 µL

human serum, employing a silica-fractionation method that separate the extract into two or four fractions for analysis.

File Name: Supplementary Data 4

Description: List of 159 sialylated GSLs identified in reference human serum along with their 3D-descriptors (*m/z*, RT, CCS).

File Name: Supplementary Data 5

Description: List of 145 neutral GSLs identified in human serum along with their 3D-descriptors (*m/z*, RT, CCS).

File Name: Supplementary Data 6

Description: List of 72 sulfatides identified in human serum along with their 3D-descriptors (*m/z*, RT, CCS).

File Name: Supplementary Data 7

Description: Internal standard partition between two SPE-GSL fractions of 6 inter-day extract replicates and results of semi-quantification strategies.

Semi-quantification strategies include:

Strategy I) the use of an average percentage of internal standard (ISTD) partition between two fractions of n = 4 extract replicate (average percentage of ISTD were calculated excluding two outliers exhibiting the anomalous ISTD partition percentages),

Strategy II) the use of percentage of ISTD partition between the two fractions in individual samples,

Combination of Strategy I and II: measurement and quantification of fraction 2 using the strategy at I) and strategy at II) only for outlier samples.

Each semi-quantification strategy has three sub-strategies pertaining to the ISTD selection and use for the GSL subclasses. The three sub-strategies (Fig. 6b) include:

1st sub-strategy: GD, O-acetyl GD, GT, GQ were normalized to GD3 18:1;O2/18:0-d3; sialylated nLc series were normalized to GM1 18:1;O2/18:0-d5; GM2 was normalized to GM2 18:1;O2/16:0-d9; GM3 was normalized to GM3 18:1;O2/18:0-d5.

2nd sub-strategy: GD, O-acetyl GD, GT, GQ, sialylated nLc series were normalized to GM1 18:1;O2/18:0-d5; GM2 was normalized to GM2 18:1;O2/16:0-d9; GM3 was normalized to GM3 18:1;O2/18:0-d5.

3rd sub-strategy: GD, O-acetyl GD, GT, GQ were normalized to GD3 18:1;O2/18:0; GM2 was normalized to GM2 18:1;O2/16:0-d9; GM3 and sialylated nLc series were normalized to GM3 18:1;O2/18:0-d5.

File Name: Supplementary Data 8

Description: Relative quantification (ng/µL serum) of the 98 identified sialylated glycosphingolipids in serum samples of controls (n = 30) and Parkinson’s Disease (n = 28) patients.

File Name: Supplementary Data 9

Description: Semi-quantification of neutral glycosphingolipids and sulfatides in serum samples of controls (n = 30) and Parkinson’s Disease (n = 28) patients.

File Name: Supplementary Data 10

Description: Results of statistical analysis using Limma method and empirical Bayesian techniques to deliver reliable differential expression predictions. Significantly different sialylated GSL between groups were identified using a 2-by-2 factorial model at an adjusted p value < 0.05 and corrected for multiple comparisons using the false discovery rate (FDR) method. We further adjusted the model for the effects of sex and age.

File Name: Supplementary Data 11

Description: Results of non-parametric Mann-Whitney U-test assessing significant differences in GSLs between control and Parkinson's Disease serum samples. The table includes the mean, standard deviation, sample size, and p-values for each comparison.
