# Supplementary material for: Extended coverage of human serum glycosphingolipidome by 4D-RP-LC TIMS-PASEF unravels association with Parkinson’s disease
Source: Nat Commun. 2025 May 16;16:4567. doi: 10.1038/s41467-025-59755-6 (PMC12084332; doi:10.1038/s41467-025-59755-6)
Supplement: Supplementary file 14 — Reporting Summary [file 41467_2025_59755_MOESM14_ESM.pdf]

nature portfolio

Corresponding author(s): Laura Bindila

Last updated by author(s): 31.01.2025

Reporting Summary

Nature Portfolio wishes to improve the reproducibility of the work that we publish. This form provides structure for consistency and transparency in reporting. For further information on Nature Portfolio policies, see our Editorial Policies and the Editorial Policy Checklist.

Statistics

For all statistical analyses, confirm that the following items are present in the figure legend, table legend, main text, or Methods section.

n/a	Confirmed	
☐	☒	The exact sample size (*n*) for each experimental group/condition, given as a discrete number and unit of measurement
☐	☒	A statement on whether measurements were taken from distinct samples or whether the same sample was measured repeatedly
☐	☒	The statistical test(s) used AND whether they are one- or two-sided<br>*Only common tests should be described solely by name; describe more complex techniques in the Methods section.*
☐	☒	A description of all covariates tested
☐	☒	A description of any assumptions or corrections, such as tests of normality and adjustment for multiple comparisons
☐	☒	A full description of the statistical parameters including central tendency (e.g. means) or other basic estimates (e.g. regression coefficient) AND variation (e.g. standard deviation) or associated estimates of uncertainty (e.g. confidence intervals)
☐	☒	For null hypothesis testing, the test statistic (e.g. *F*, *t*, *r*) with confidence intervals, effect sizes, degrees of freedom and *P* value noted<br>*Give P values as exact values whenever suitable.*
☒	☐	For Bayesian analysis, information on the choice of priors and Markov chain Monte Carlo settings
☒	☐	For hierarchical and complex designs, identification of the appropriate level for tests and full reporting of outcomes
☒	☐	Estimates of effect sizes (e.g. Cohen's *d*, Pearson's *r*), indicating how they were calculated

Our web collection on statistics for biologists contains articles on many of the points above.

Software and code

Policy information about availability of computer code

Data collection	The instrumental calibrations and the data acquisition were controlled by Compass Hystar 6.3 and timsControl 5.0.
Data analysis	The identification of the glycosphingolipids was performed using Metaboscape 2021b and Compass DataAnalysis 6.1. A 4D library for glycolipids was generated in Metaboscape 2021b from data acquired from standard GSLs , commercially available mixtures of GSL, and GSLs extracted from sera of volunteers. The hence annotated GSL descriptor values were used to generate a target list for data processing using Skyline 23.1.0.280, e.g. determination of peak areas from extracted ion chromatograms of individual GSLs for quantification purpose. The peak areas of GSLs were then exported to the MS Excel 2016 and all the quantification were perform therein. All the visualizations and statistics were produced using MS Excel 2016, GraphPad Prism 9.5.1, Origin 2022, CorelDraw 2022 V5, and R.

For manuscripts utilizing custom algorithms or software that are central to the research but not yet described in published literature, software must be made available to editors and reviewers. We strongly encourage code deposition in a community repository (e.g. GitHub). See the Nature Portfolio guidelines for submitting code & software for further information.

Data

Policy information about availability of data

All manuscripts must include a data availability statement. This statement should provide the following information, where applicable:

- Accession codes, unique identifiers, or web links for publicly available datasets
- A description of any restrictions on data availability
- For clinical datasets or third party data, please ensure that the statement adheres to our policy

The raw data of reference samples used for method development and GSL spectral libraries: for ganglio-, neolacto- and neutral GSLs and sulfatides generated in this study have been deposited in the MASS Spectrometry Interactive Virtual Environment (MassIVE) under the identifier MSV000097015 [https://dx.doi.org/10.25345/C53J39C93]. The spectral library generated and used in this study is additionally available under the following link: Research Tools [https://www.unimedizin-mainz.de/lipidomics-unit/lipid-research/research-tools.html]. Unless otherwise stated, all processed data supporting the results of this study can be found in the article, supplementary data, and source data files. As recommended by the International Lipidomics Society, the reporting checklist from the Lipidomics Standards Initiative is included in the Supplementary Materials. Source Data are provided with this paper.

Research involving human participants, their data, or biological material

Policy information about studies with human participants or human data. See also policy information about sex, gender (identity/presentation), and sexual orientation and race, ethnicity and racism.

Reporting on sex and gender	Sera from two volunteer co-authors; Gender: Female<br>Sera from 30 healthy control (14 females and 16 males) and 28 Parkinson’s Disease (12 females and 16 males).
Reporting on race, ethnicity, or other socially relevant groupings	The sera from volunteers was exclusively used for method development and method's performance evaluation.<br>The serum samples used for the proof-of-concept and method's applicability for clincial phenotype study were obtained from two groups: control individuals who donated their serum, and Parkinson's disease patients whose diagnosis was confirmed by the Movement Disorders, Imaging and Neurostimulation Department of Neurology at the University Medical Center Mainz in Germany. Demographic data on race, identity, gender, sexual orientation and ethnicity were not included in the statistical analysis.
Population characteristics	Sera from two volunteer co-authors: None of the participants had made any changes in their usual lifestyle, and did not experienced any symptoms nor was diagnosed suffering from infectious disease during the study. No data were collected regarding genotype or past diagnoses/treatments. No clinical data were collected and used in any form for this study.<br>Sera from 30 healthy control and 28 Parkinson’s Disease: The population ranged from 24 to 80 years.
Recruitment	Sera donated by two co-authors of the manuscript was used to optimize and evaluate method and generate serological GSL library.<br>Sera of Parkinson’s Disease patients (n = 28) along with age-matched control sera (n = 30) were collected by Movement Disorders, Imaging and Neurostimulation, Department of Neurology, University of Medical Center Mainz, Germany and used for proof-of-concept of the methodology.
Ethics oversight	Sera of two co-author volunteers were used for extraction method and GSL library development and proof-of-concept for 4D PASEF workflow. All two co-authors received information on study specific requirements for participation and signed corresponding declaration of consent. This proof-of-concept study did not fall under the responsibility of Ethical Commission.<br>For the method's application evaluation, the comparison of sera GSL from controls and Parkinson patients was undertaken, whereby sera samples from Parkinson’s Disease patients (n = 28) along with age-matched control sera (n = 30) were used. The study participants provided an informed consent and the study was approved with project Nr. 837.311.12(842-F) by the Ethical Committee of the University Medical Center of Mainz, Rheinland Pfalz, Germany.

Note that full information on the approval of the study protocol must also be provided in the manuscript.

Field-specific reporting

Please select the one below that is the best fit for your research. If you are not sure, read the appropriate sections before making your selection.

☒ Life sciences ☐ Behavioural & social sciences ☐ Ecological, evolutionary & environmental sciences

For a reference copy of the document with all sections, see nature.com/documents/nr-reporting-summary-flat.pdf

Life sciences study design

All studies must disclose on these points even when the disclosure is negative.

Sample size	For analytical workflow development and assessment, NIST 1951c SRM and two co-author volunteers sera were used. Six replicate extractions were prepared and used for evaluation of partition coefficient of glycolipid internal standrads between two fractions. Six replicate extraction of reference sera rendering 2 individual fractions were prepared for intra-assay evaluation. Inter-assay performance was evaluated based on 2 replicate extracts of n=3 sera samples. The n=6 replicates was deemed sufficient to properly evaluate method`s reproducibility and robustness

	particularly for sample preparation procedure including multiple steps of fractionations and chromatographic purification and enrichment. For proof-of-concept application: control (n = 30) and Parkinson's Disease (n = 28) serum samples were used. This number was considered sufficiently representative for a pilot study, using samples from a single clinical center, to draw solid insights into serological GSL profile in Parkinson's disease.
Data exclusions	In calculation of the average of the percentage partition of internal standard between two fractions in 6 extract replicates, two outliers were excluded since two extraction replicates exhibited anomalous internal standard partitions.
Replication	For establishing GSL library and assessment of analytical performance of 4D-tims-PASEF method, all experiments were run in replicates n >= 3. 6 inter-day extract replicates of volunteer serum were used to test the analytical workflow. Quality of the annotation score shows good analytical performance of the method and the reproducibility of the workflow is reflected with the CV of less than 35% for most of the demonstrated sialylated glycosphingolipids. For proof-of-concept application, each sample from the controls and Parkinson was extracted and measured once in each positive and negative ion mode respectively.
Randomization	All data from proof-of-concept study were analyzed in a single batch. Extraction of GSL from samples was parallelized with random order of the sample extraction. Data of the proof-of-concept study were processed in Metaboscape and Skyline at once in a single bucket and batch and data feature extraction and processing was independent of sample groups. Randomisation of the sample processing within a sample group was invariably performed at every stage of the workflow.
Blinding	The analytical scientist was blinded to any clinical data and/or values of the human serum samples from controls and PD used in the proof-of-concept application. All data were processed in Metaboscape at once in a single bucket and batch and data feature extraction and processing was/is independent of sample groups.

Reporting for specific materials, systems and methods

We require information from authors about some types of materials, experimental systems and methods used in many studies. Here, indicate whether each material, system or method listed is relevant to your study. If you are not sure if a list item applies to your research, read the appropriate section before selecting a response.

Materials & experimental systems

n/a	Involved in the study	
☒	☐	Antibodies
☒	☐	Eukaryotic cell lines
☒	☐	Palaeontology and archaeology
☒	☐	Animals and other organisms
☒	☐	Clinical data
☒	☐	Dual use research of concern
☒	☐	Plants

Methods

n/a	Involved in the study	
☒	☐	ChIP-seq
☒	☐	Flow cytometry
☒	☐	MRI-based neuroimaging

Plants

Seed stocks	*Report on the source of all seed stocks or other plant material used. If applicable, state the seed stock centre and catalogue number. If plant specimens were collected from the field, describe the collection location, date and sampling procedures.*
Novel plant genotypes	*Describe the methods by which all novel plant genotypes were produced. This includes those generated by transgenic approaches, gene editing, chemical/radiation-based mutagenesis and hybridization. For transgenic lines, describe the transformation method, the number of independent lines analyzed and the generation upon which experiments were performed. For gene-edited lines, describe the editor used, the endogenous sequence targeted for editing, the targeting guide RNA sequence (if applicable) and how the editor was applied.*
Authentication	*Describe any authentication procedures for each seed stock used or novel genotype generated. Describe any experiments used to assess the effect of a mutation and, where applicable, how potential secondary effects (e.g. second site T-DNA insertions, mosiacism, off-target gene editing) were examined.*
